# Work-Related Stressors Among Maternal, Infant, and Early Childhood Home Visiting (MIECHV) Home Visitors: A Qualitative Study

**DOI:** 10.1007/s10995-018-2536-8

**Published:** 2018-05-31

**Authors:** Paige J. Alitz, Shana Geary, Pamela C. Birriel, Takudzwa Sayi, Rema Ramakrishnan, Omotola Balogun, Alison Salloum, Jennifer T. Marshall

**Affiliations:** 1 Department of Community and Family Health, College of Public Health, University of South Florida, Tampa, FL USA; 2 Department of Epidemiology and Biostatistics, College of Public Health, University of South Florida, Tampa, FL USA; 3 School of Social Work, College of Behavioral and Community Sciences, University of South Florida, Tampa, FL USA

**Keywords:** Home visitation, Burnout, Work-related stress, Coping mechanisms, Social support

## Abstract

*Background* The Florida Maternal, Infant, and Early Childhood Home Visiting (MIECHV) program delivers evidence-based home visiting services to over 1400 families each year. Home visitors are integral in providing resources for families to promote healthy pregnancy, child development, family wellness, and self-sufficiency. Due to the nature of this work, home visitors experience work-related pressures and stressors that can impact staff well-being and retention. *Objectives* The purpose of this study was to understand primary sources of work-related stress experienced by home visitors, subsequent effects on their engagement with program participants, and to learn of coping mechanisms used to manage stress. *Methods* In 2015, Florida MIECHV program evaluators conducted ten focus groups with 49 home visitors during which they ranked and discussed their top sources of work-related stress. Qualitative analysis was conducted to identify emergent themes in work-related stressors and coping/supports. *Results* Across all sites, the burden of paperwork and data entry were the highest ranked work-related stressors perceived as interfering with home visitors’ engagement with participants. The second-highest ranked stressors included caseload management, followed by a lack of resources for families, and dangerous environments. Home visitors reported gratification in their helping relationships families, and relied on coworkers or supervisors as primary sources of workplace support along with self-care (e.g. mini-vacations, recreation, and counseling). *Conclusions for practice* Florida MIECHV home visitors across all ten focus groups shared similar work-related stressors that they felt diminished engagement with program participants and could impact participant and staff retention. In response, Florida MIECHV increased resources to support home visitor compensation and reduce caseloads, and obtained a competitive award from HRSA to implement a mindfulness-based stress reduction training statewide.

## Significance

It is well known that home visitors balance strenuous caseloads that include families facing complex social and health related problems, but little is known about specific work-related stressors that impact staff and affect family engagement within evidence-based home visiting programs. This study identifies sources of staff stress within the Florida MIECHV program and home visitors’ perceptions around how that stress directly impacts engagement with participants. A better understanding of these concepts will help programs identify effective methods to mitigate home visitor stress to ultimately improve program effectiveness.

## Introduction

Maternal and child home visitors provide a specialized set of supports and resources to families using various program models and curricula (Gomby 2005; Sweet and Appelbaum 2004). While research shows that home care workers experience stress due to heavy caseloads, difficult clients, and safety hazards in client homes, there is scant literature on work-related stressors specifically among home visiting staff in an evidence-based program (Denton et al. 2002). The multifaceted responsibilities of home visitors in evidence-based programs contribute to work-related stress: delivering a specific curriculum; addressing multiple social determinants of health; documenting their efforts; and continuous professional development (Barak et al. 2014; Gill et al. 2007; Williams et al. 2008). Federal agencies increasingly support evidence-based models which promote rigorous evaluation of health outcomes; allocating funds to programs that make strides towards benchmark indicators (Boller et al. 2010). Sharp et al. (2003) suggested that while evidence-based models may provide more consistency and structure in program delivery, a focus on outcome measures may divert attention from some aspects of the program that may mediate these outcomes, like home visitor–parent relationships (Brookes et al. 2006; Dunst et al. 2002). Administrative burden on staff working in evidence-based programs could also be higher.

**Fig. 1:**
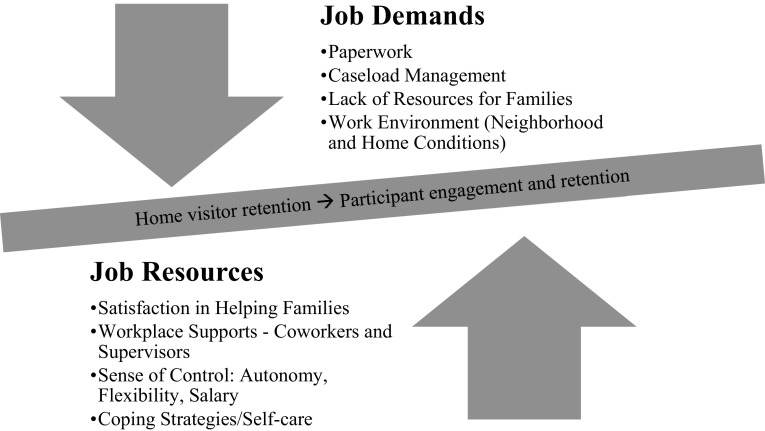
Themes identified related to work-related stressors and coping strategies

Because work-related stress can lead to burnout, reducing the quality of home visiting services, and staff turnover inhibiting client engagement and, identifying sources of these stressors among home visitors is imperative (Dickinson and Perry 2002; Khamisa et al. 2015; Lee et al. 2013; Maslach et al. 2001). This qualitative study explored the perceptions of home visitors in Florida’s evidence-based maternal, infant, and early childhood home visiting (MIECHV) programs regarding work-related stressors and coping strategies, and the potential impacts on engagement with participants and participant and staff retention. The Job Demands-Resources Model recognizes that high-demand jobs “that require sustained physical and/or psychological (cognitive and emotional) effort or skills…are therefore associated with certain physiological and/or psychological costs” which interact with personal and organizational resources, impacting motivation and causing job strain (Bakker and Demerouti 2007, p. 312; see Fig. 1).

The Florida MIECHV initiative supports the coordinated implementation of three evidence-based home visiting program models: parents as teachers, nurse-family partnership, and healthy families Florida. Across models, the maximum caseload per Florida MIECHV home visitor is 25 and the expectation is that each family receives two home visits per month. The statewide MIECHV evaluation team is housed in the Chiles Center at the University of South Florida (USF), and the evaluation has been determined exempt by the USF Institutional Review Board. As part of the evaluation, the purpose of this study was to understand the primary sources of work-related stress experienced by Florida MIECHV home visitors, how these stressors affected their engagement with participants, and the coping mechanisms home visitors used to mitigate work-related stress. Given that work-related stress may impact the retention of home visitors and program participants, staff were asked about their perceptions regarding how work-related stressors impact staff and participant retention.

## Methods

In Fall 2015, ten focus groups were held with Florida MIECHV home visitors on-site at each of the ten MIECHV local implementing agencies throughout the state. The 60–90 min group discussions were led by a trained facilitator and an assistant. Topics included general questions about the program, needs of families served, sources of work-related stress encountered, and coping strategies commonly used by home visitors to mitigate these stressors.

Specific questions within the focus group guide that related to stress included: What are some of the main sources of stress among home visitors? Aside from those listed, can you think of any other sources of stress among home visitors? How do you think this affects staff recruitment and retention? How do you think this affects work with families? What supports are available to home visitors in this program? and What other coping/support strategies do home visitors use to deal with work-related stress? Additionally, a pile sorting activity was conducted in which participants were provided five notecards and instructed to write one work-related stressor on each card. The group then sorted the notecards into clusters of related items and lined them up sequentially from the highest to the lowest contribution to work-related stress. The reason for using notecards was threefold. Firstly, the research team anticipated that participants may feel more comfortable writing potentially sensitive stressors on cards placed in a pile without having to directly state them in a group. Secondly, listing ideas on notecards facilitated brainstorming, which is important given the tendency of focus group discussions to follow conversational paths that may limit topics discussed. Third, ranking and grouping the cards helped participants articulate and describe why some stressors were more salient to them.

All ten focus groups were audio recorded, professionally transcribed verbatim, and reviewed by research staff for accuracy. Preliminary inductive analysis identified emergent themes from the transcripts, then coding was performed electronically with MAXQDA Version 11 (VERBI GmbH, 1989–2014) following a codebook developed by the research team based on the research questions, focus group guide, and emergent themes. A lead analyst coded each of the ten focus group transcripts, while a second analyst blind-coded three of the ten transcripts. The kappa statistic was 0.78 (95% confidence interval: 0.73, 0.83) indicating substantial inter-coder agreement. Descriptive statistics were calculated from self-reported demographics entered into Qualtrics Survey Software (2015) and stored on a secure server.

## Results

Ten focus groups were conducted with 49 MIECHV home visitors (Table 1). About a third of the home visitors were under the age of 35 (34.7%), and most had worked in their current position for less than five years (84.0%). Most (87.8%) held an undergraduate or graduate college degree in nursing (36.7%), more than one discipline (20.4%), or social work (14.3%). Over half of the home visitors self-identified as non-Hispanic (69.4%), were White (53.1%), and lived in the communities in which they served (61.2%). Job demands (stressors) and resources (coping) are illustrated in Fig. 1.

**Table 1:** Frequency distribution of home visitor demographics

Demographics	N = 49 (%)
Number of years in current position	
< 1 year	24 (49.0)
1–5 years	17 (34.7)
6–10 years	4 (8.2)
> 10 years	3 (6.1)
Prefer not to answer	1 (2.0)
Education	
High school degree	1 (2.0)
Some college	5 (10.2)
College degree (associates/bachelors)	35 (71.4)
Graduate degree (masters/doctoral)	8 (16.3)
Professional background	
Nursing	18 (36.7)
More than one discipline	10 (20.4)
Social work	7 (14.3)
Psychology/counseling	4 (8.2)
Other^a^	4 (8.2)
Education	3 (6.1)
Public health	1 (2.1)
Prefer not to answer	2 (4.1)
Age, years	
35+	32 (65.3)
30–34	7 (14.3)
25–29	7 (14.3)
20–24	3 (6.1)
Race	
White	26 (53.1)
Black	16 (32.7)
Other	6 (12.2)
Prefer not to answer	1 (2.1)
Ethnicity	
Hispanic	14 (28.6)
Non-Hispanic	34 (69.4)
Prefer not to answer	1 (2.1)
Live in community served by the program	
Yes	30 (61.2)
No	18 (36.7)
Prefer not to answer	1 (2.1)

^a^Other includes social justice, business administration, health care administration, criminal justice, international studies, and communication

### Job Demands

#### Work-Related Stressors

##### Management of Paperwork

In seven of the ten sites, home visitors expressed that sometimes-required documentation interfered with their ability to optimally engage with participants during their visits. One home visitor explained that excessive paperwork “really puts a barrier and monkey wrench” in their visits. Furthermore, home visitors in eight of the ten sites felt that their personal connection with families was not given the same level of importance as the outcome data captured through required documentation. As one home visitor said:

Unfortunately, the funders are not there to see, “Hey, you have a pregnant mom with twins who’s afraid to go out, and you manage to get this lady to get a job, to get her child into daycare”…What the funder is seeing is, “Are those women going to the hospital, how many times are they going to the ER? Are they going to the ER less? Are they up-to-date with immunization?” That’s what they care about, and that’s the difference.

##### Caseload Management

In nine of ten sites, home visitors felt that travel time and other responsibilities associated with managing a caseload encroached on the time needed to engage with families. One home visitor expressed frustration with the hurriedness of her case schedule: “…if a mom needs me to stay an extra 30 min to talk, I can’t because I got another visit, I got to be there in 30 min, so I can’t help you right now.” Depending on the program model, each home visitor may be scheduled to see 20–25 clients every other week, or more frequently. The challenge was not so much the caseload size as the instability and frequency of crises among this high-risk population. As one home visitor put it, “that’s 25 problems, 25 people to try to help them in everything.”

Nearly all groups discussed how families cancel or reschedule frequently, often when they are already en route, contributing to a cyclical scheduling problem and creating additional pressure on the home visitor, who could have used the time for other work responsibilities:

This is time that you can give to another person. It is time that you can utilize working in the office. It’s a waste of time. You have too many things to do, too many visits to accomplish…you already drive 30 minutes, 10 minutes to get there. Knock on the door, she’s not there. One hour you waste that you can use on something else.

##### Lack of Resources for Families

Home visitors discussed difficulties in finding services for families like housing, childcare, and transportation, especially in rural MIECHV sites. One home visitor mentioned an 18-month waiting list for childcare in their community. This situation aggravates a vicious cycle of not being able to work, and thus afford housing or other bills—a common scenario that contributes to home visitors’ stress. The lack of mental health services, and long waiting lists for available services, were additional concerns expressed by many home visitors, because they are not trained as mental health providers. As one home visitor described:

I have my clients who–while referred to the [agency name] program, she was on waiting list and nobody called her. And a few weeks later she called me and she told me, “I feel like killing myself.” Who was there? So, whenever she feels depressed…10:00, 11:00 at night, who she calls? Me, while we’re waiting for [agency] to call her back.

##### Dangerous Environments

The home visitors consistently expressed a passion for supporting families living in high-need communities, but described the stress of encountering drug dealing, crime, and gun violence in the participants’ neighborhoods. One home visitor recalled when a client’s neighbors was shot in front of her house. In addition to concerns with neighborhood safety, home visitors noted risks within some client’s homes, mentioning that often they do not know what they are “walking into” when they stop by: “If they’ve forgotten that we’re coming, we also don’t want to walk into a bad situation where we’re not invited.”

#### Impacts on Home Visitor-Participant Engagement

Home visitors were explicit about their skill in suppressing personal stress when engaging directly with families, though it takes an emotional toll. As explained by one home visitor, “I could be crying now and then I’ll go to my clients and whatever and then I leave—but what that makes me is more burn out, more stressed.” Said another,

When I go to visit, it’s about them. It’s not about what happened to me or how hard it is for me to do my job or whatever. It’s just about being there for them and whatever they need from me. But of course, you’re frustrated, and it is very hard.

Filling out paperwork/assessments during home visits was referenced as the primary work-related stressor interfering with home visitors’ engagement with families during visits. One home visitor explained:

…this is my plan today, and then mom starts talking about something personal and then [I’m] listening to her I’m thinking, “oh my God, I need to do the ASQ [Ages & Stages Questionnaire]” and she’s still talking. I need to do the ASQ and I need to be leaving soon because then I have another mom to see. I’m not even focusing on her!

The ability of home visitors to engage with participants is also affected when rescheduling and cancellations occur. Without meeting frequently, the level of contact needed for the home visitor to build a trusting relationship, while effectively delivering a curriculum and services that can impact positive health outcomes, is not achieved.

Managing caseloads and family engagement was also difficult for home visitors who attend beneficial, yet time-consuming, meetings and conferences for professional development. During one focus group, a home visitor explained feeling like she “had to rush sometimes with [my] clients, especially when they’re in a crisis…” due to other job-related obligations. Another home visitor spoke about visiting a client in crisis, but because there was a required meeting to attend, this home visitor had to leave in the middle of the woman’s emotional breakdown.

#### Impacts on Participant Retention

Schedule changes impact engagement, and subsequently retention of participants, as one home visitor explained, “…because unfortunately, cancellations lead to disengagement, disengagement leads to low numbers, our numbers drop.” Additionally, home visitors mentioned how completion of required paperwork can intimidate clients into being less willing to proceed with the program. For example, there was one instance where a client commented on the number of pages on the intake form; the home visitor halted the visit in fear of losing the client before their first meeting was over, noting how losing clients “happens a lot,” because “they’re probably thinking, ‘if we had to do this on day one, God only knows what they’ll have me doing every day.’”

The lack of resources available in the community was perceived to impact participant retention in some MIECHV programs more than others. In one community, a home visitor felt as an “essential” part of participants’ lives; even if home visitors could not connect them with transportation or another resource, the client depended on them for the personal relationship. In some communities, home visitors perceived that some families joined the program solely to obtain needed supplies such as cribs, federal aid money, or car seats. This motive inherently affected client retention because those participants saw home visitors as a “go-between… between the different types of services that they need…” These families would leave the program once material needs were met.

#### Impacts on Home Visitor Retention

Staff turnover varied between sites, with some experiencing high turnover while other sites had very little; one site described their staffing as “solid.” One home visitor explained:

…for me to recommend this job to someone I would have to know them very well. I would have to know that they’re organized. I would have to know some things about them before I would encourage them…. I wouldn’t tell them to take this job just because they need a job. This is not the job you take just because you need a job.

### Job Resources

#### Satisfaction in Helping Families

Home visitors across all MIECHV sites expressed how helping and building relationships with families and seeing the positive changes in response to their efforts was the most gratifying aspect of their job, as one described, “Being able to help the families. Point them in the right direction where they need to go to get the help that they need.” Home visitors also felt satisfaction in watching their clients become more independent, securing jobs, and following through with the referrals given to them.

…you make a referral because you know they probably need it and they agree to it then they might not follow through and then you’re waiting and waiting but they eventually do. So, that progress that they have as well with their baby developing and the fact we’re there helping them with letting them know how the baby should be developing and stuff. That does really make you feel good about your job.

#### Workplace Supports: Coworkers and Supervisors

The home visitors consistently identified each other as their greatest form of support in dealing with the work-related stressors. In seven out of ten sites, supervisors were mentioned as another form of support. These home visitors noted how the use of reflective supervision allowed them to vent their frustrations, express their feelings, and talk freely about how their job affects them personally. As one home visitor described of their site supervisor: “She’ll always say, ‘Is there anything I can do for you? How can I help you?’” Conversely, at one site, the lack of support from coworkers and supervisors was damaging. A few home visitors spoke of their unhappiness and stress over their job-related duties, feeling as though they had no one to turn to when they needed help with a family. In one site that was undergoing a transition in leadership, a home visitor stated, “we don’t know how to work with each other anymore.”

#### Sense of Control: Autonomy, Flexibility, Salary

Although not explicit in most discussions, home visitors’ need for a sense of control over their work demands was implicit in many comments. They conveyed commitment, satisfaction, and confidence in working with families but often expressed frustration at their lack of control over their schedules, due to client cancellations or staff meetings and to balancing programmatic demands with family needs. The sentiments reflected a lower sense of control over their job demands and the subsequent strain, as one home visitor shared: “So, you feel this pressure like, I can’t change my situation. If you can’t change your situation, you’re like, ‘Why should I be here?’ Because I’m doing all I can.” Also, in some groups, there was discussion regarding differential rates of pay across sites and home visiting models—and even within one program that was implemented by two organizations. Some home visitors explained that they could make a higher hourly rate in other types of positions, and among staff in some programs there was concern about the low rate of pay overall. Salaried positions offered more scheduling flexibility.

#### Coping Strategies

Home visitors cited the use of exercise and meditation techniques such as yoga, dance, and mindfulness practices to ease their work-related stress. These activities were self-directed, and sometimes encouraged and supported by the employer. Oftentimes these strategies were utilized during employee retreats; supervisors provided time off for home visitors to attend group classes or mindfulness seminars. Home visitors also spoke of the stress-relieving effects of spending time with their own children and families; one reflected that taking her sister’s grandchildren on outings (restaurant/arcade, swimming, bike riding, etc.) was the “greatest thing in the world” for stress management.

## Discussion

This study identified job demands and resources contributing to home visitors’ overall job stress and satisfaction. The highest reported source of stress was paperwork/documentation required by both MIECHV and their respective program models, along with the requisite caseload and number of visits, lack of resources for families, and unsafe environments. These factors negatively influenced engagement by diluting the highly valued relationships between home visitors and families, and could ultimately impact retention of staff and participants.

The notion that a strict focus on documenting and achieving program outcomes conflicts with the home visitor-client relationship can be found elsewhere in the literature (Barak et al. 2014; Brookes et al. 2006). A study of 85 Illinois home visitors from three evidence-based models found that, while the home visitors appreciate the need for program fidelity, the amount of paperwork required undermined the importance of their relationships with clients (Barak et al. 2014). Much like those in our study, the Illinois home visitors felt that their clients were being reduced to quantifiable data points denoted as “numbers” and “results” rather than seen as human beings facing and overcoming everyday obstacles, and worried that paperwork required during each home visit interfered with the natural course of relationship building with their clients, diminishing the client-centered nature of the program (Barak et al. 2014).

The balance between training/professional development, salary and benefits, and supportive work environment with job demands impacts home visitors’ job satisfaction and burnout (Gill et al. 2007). To balance the demands of home visiting as a profession, home visitors primarily relied on organizational resources (e.g., social support from coworkers and supervisors, reflective supervision) and various modes of self-care. Prior research also suggests that quality supervision and a high level of support from colleagues contribute to the effectiveness of home visitors (Gill et al. 2007; Wasik and Bryant 2001). A change in leadership has been identified as a period where home visitors may feel the most stressed and least satisfied with their jobs (Gill et al. 2007) as was evident in one program site in this study.

Home visiting as a profession requires a unique set of knowledge, attitudes, and skills in health and safety, mental health, and learning; assessment, reflective practice and supporting families/parenting; leadership, diversity/inclusion, and professionalism (Roggman et al. 2016). Training for home visitors should incorporate guidance on how to simultaneously manage their role as a support system for the families they serve while executing program requirements. While the burden of paperwork contributes to work-related stress in evidence-based programs, assessments and documentation are vital components; more efficient methods for data collection would save time, and better conveyance of the ultimate benefits of evidence-based programs to families could reduce dissonance. In addition to the logistics of caseload management, training and support for handling the emotional labor involved in home visiting while capitalizing on the satisfaction home visitors express in helping families can reduce stress and burnout (Brotheridge and Grandey 2002; Humphrey et al. 2015).

The results of this study, conducted with Florida MIECHV staff may not be generalizable to MIECHV programs in other states. The experiences of other home visitors in Florida who are not within MIECHV (funded under different structures and reporting requirements) may also reflect different or additional stressors, supports, and coping methods. We also note that almost half of the home visitors who participated in this study were within their first year of working in the MIECHV program; stressors among home visitors working in a newly funded program, or those who are new to the profession, likely differ compared to those who are more experienced and may be more adept at or better equipped to manage program requirements.

## Conclusions

Home visitors play a vital role in promoting positive outcomes for children and families. Programmatic efforts to mitigate work-related stress could increase the well-being, effectiveness, and consistency of home visitors. Following this study: Florida MIECHV increased resources to sites specifically to increase home visitor compensation, hire data entry staff, and reduce caseloads where needed. Additionally, a competitive grant from the Health Resources and Services Administration was awarded to implement a mindfulness-based stress-reduction program for home visitors statewide; and reflective supervision training is ongoing. More research is needed to compare stressors and coping among newer versus more seasoned home visitors, to understand the benefits and toll of emotional labor on home visitors, and to further dissect the intersection between caseloads, required documentation, and supporting families in evidence-based home visiting.
