# Correction to: Work-Related Stressors Among Maternal, Infant, and Early Childhood Home Visiting (MIECHV) Home Visitors: A Qualitative Study

**DOI:** 10.1007/s10995-018-2622-y

**Published:** 2018-08-22

**Authors:** Paige J. Alitz, Shana Geary, Pamela C. Birriel, Takudzwa Sayi, Rema Ramakrishnan, Omotola Balogun, Alison Salloum, Jennifer T. Marshall

**Affiliations:** 1 Department of Community and Family Health, College of Public Health, University of South Florida, Tampa, FL USA; 2 Department of Epidemiology and Biostatistics, College of Public Health, University of South Florida, Tampa, FL USA; 3 School of Social Work, College of Behavioral and Community Sciences, University of South Florida, Tampa, FL USA

## Correction to: Maternal and Child Health Journal 10.1007/s10995-018-2536-8

The article “Work-Related Stressors Among Maternal, Infant, and Early Childhood Home Visiting (MIECHV) Home Visitors: A Qualitative Study”, written by Paige J. Alitz, Shana Geary, Pamela C. Birriel, Takudzwa Sayi, Rema Ramakrishnan, Omotola Balogun, Alison Salloum and Jennifer T. Marshall, was originally published electronically on the publisher’s internet portal (currently SpringerLink) on 31 May 2018 without open access. With the author(s)’ decision to opt for Open Choice the copyright of the article changed on 25 July 2018 to © The Author(s) 2018 and the article is forthwith distributed under the terms of the Creative Commons Attribution 4.0 International License (http://creativecommons.org/licenses/by/4.0/), which permits use, duplication, adaptation, distribution and reproduction in any medium or format, as long as you give appropriate credit to the original author(s) and the source, provide a link to the Creative Commons license and indicate if changes were made.

The original article has been corrected.

